# Partial tarsal arthrodesis with plate fixation for management of traumatic tarsometatarsal injuries in five cats

**DOI:** 10.1111/jsap.70077

**Published:** 2025-12-18

**Authors:** R. L. Nixon, D. N. Clements, J. M. Ryan

**Affiliations:** ^1^ The Royal (Dick) School of Veterinary Studies, Hospital for Small Animals The University of Edinburgh Midlothian UK

## Abstract

**Objectives:**

To describe the aetiology, presentation, complications and outcome in cats with traumatic injuries of the tarsometatarsal joint treated with partial tarsal arthrodesis.

**Materials and Methods:**

Retrospective series of five client‐owned cats.

Medical records were retrieved for cats with traumatic injuries of the tarsometatarsal joint treated with partial tarsal arthrodesis between November 2011 and December 2024 at a veterinary teaching hospital. Sex, age at the time of surgery, presenting lameness, surgical technique used, follow‐up time, complications and outcome were recorded for each cat.

**Results:**

Five cats were included in this study; of these, four developed the injury as a result of catching their paw in a horizontal drawer handle. All cats were treated with partial tarsal arthrodesis stabilised with a bone plate, applied laterally in three cats and medially in two cats. No intra‐operative complications or major post‐operative complications were recorded. Four of the five cats had post‐operative paw swelling on the operated limb. Clinical and radiographic follow‐up assessment was performed 60 to 87 days post‐operatively. At final recheck examination, four of the five cats exhibited no lameness. One cat exhibited mild weight bearing lameness, and one cat had mild iatrogenic tarsal valgus associated with incorrect plate contouring.

**Clinical Significance:**

Partial tarsal arthrodesis with bone plate fixation for management of traumatic injury of the tarsometatarsal joint in cats is associated with excellent outcomes for return to pre‐injury activities. The procedure commonly results in transient paw swelling in the immediate post‐operative period. Horizontal drawer handles pose an injury risk to cats jumping from heights.

## INTRODUCTION

Tarsometatarsal hyperextension or subluxation occurs due to damage to the plantar fibrocartilage, plantar supporting ligaments and collateral ligaments as a result of trauma, degeneration or congenital abnormalities (Harasen, 2002; Murakami et al., 2023; Yardımcı et al., 2016). In dogs, tarsometatarsal instability accounts for 12% of hock injuries (Campbell et al., 1976). The frequency of tarsometatarsal instability in cats is unknown.

Tarsometatarsal arthrodesis is generally recommended for the treatment of canine tarsometatarsal instability (Harasen, 2002) as non‐surgical management (Arwedsson, 1954) including the use of external coaptation is associated with poorer outcomes (Campbell et al., 1976). Partial tarsal arthrodesis (ParTA) is an orthopaedic salvage procedure, which results in the bony fusion of the tarsometatarsal joint, proximal intertarsal or distal intertarsal or a combination thereof (Chow & Balfour, 2012; Inauen et al., 2009). A variety of stabilisation methods for feline ParTA are reported. Four techniques have been reported for stabilisation of feline partial tarsal arthrodesis for injuries to the tarsometatarsal joint: (1) the use of three Kirschner wires (K‐wires) (Chow & Balfour, 2012), (2) the use of four K‐wires (Chow & Balfour, 2012), (3) stabilisation using a dorsally applied bone plate (Inauen et al., 2009) and (4) biaxial plating (Philips & Jerram, 2025).

This case series describes the aetiology, presentation, complications and outcome following partial tarsal arthrodesis with medial or lateral bone plate fixation in five cats for the management of traumatic injuries to the tarsometatarsal joint.

## MATERIALS AND METHODS

### Study population

Medical records were retrieved for cats which had undergone partial tarsal arthrodesis for the management of traumatic tarsometatarsal injuries between November 2011 and December 2024 at a veterinary teaching hospital.

Inclusion criteria included the availability of information and radiographs related to the pre‐operative and immediate post‐operative time periods as well as follow‐up assessment 8 to 13 weeks post‐operatively. The sex, age at the time of surgery, presenting lameness, surgical technique used, follow‐up time, complications and outcome for each cat were recorded. Complications were classified as catastrophic, major and minor according to Cook et al. (2010).

### Radiographic assessment

**FIG 1:**
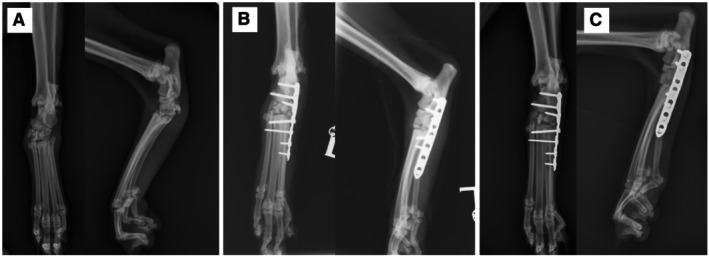
Partial tarsal arthrodesis of Case 1, a 15‐year 4‐month male neutered domestic shorthair cat (A) Pre‐operative radiographs show dorsal subluxation of the tarsometatarsal joint and widening of the medial aspect of the tarsometatarsal joint. A small bony fragment is visible dorsal to the tarsometatarsal joint, suspected to be a small chip fracture. (B) Immediate post‐operative mediolateral and dorsoplantar radiographs of the left tarsus following partial tarsal arthrodesis stabilised with a laterally applied seven‐hole 1.5/2.0 mm locking compression plate with two 2.0 mm locking screws, two 2.0 mm cortical screws and three 1.5 mm cortical screws. (C) Follow‐up radiographs taken 65 days post‐operatively showing progressive ankylosis of the partial tarsal arthrodesis.

**FIG 2:**
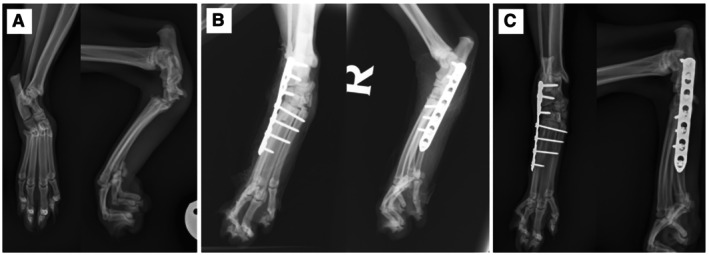
Partial tarsal arthrodesis of Case 2, a 2‐year 4‐month female neutered domestic shorthair cat (A) Pre‐operative radiographs show dorsal subluxation of the tarsometatarsal joint and widening of the medial aspect of the tarsometatarsal joint. A small bony fragment is visible proximodorsal to the tarsometatarsal joint, suspected to be a small chip fracture. (B) Immediate post‐operative mediolateral and dorsoplantar radiographs of the right tarsus following partial tarsal arthrodesis stabilised with a laterally applied seven‐hole 1.5/2.0 mm locking compression plate with two 2.0 mm locking screws, one 2.0 mm cortical screw and four 1.5 mm cortical screws. Tarsal valgus is present. (C) Follow‐up radiographs taken 77 days post‐operatively showing progressive ankylosis of the partial tarsal arthrodesis and tarsal valgus.

Pre‐operative mediolateral and dorsoplantar radiographs of the tarsus were evaluated to assess the level of instability and the presence of additional fractures with stress views if indicated (see Figs 1 and 2A). Immediate post‐operative radiographs of the tarsus were taken to assess implant positioning, alignment and reduction (see Figs 1 and 2B). Follow‐up radiographs were taken 8 to 12 weeks post‐operatively (see Figs 1 and 2C). Orthogonal radiographs from the final recheck examination were scored on a scale from 0% to 100% to describe the extent of the osseous bridging as described by Longo et al. (2025).

### Surgical procedure

Anaesthesia protocol was at the discretion of the attending anaesthetist. Four of the five cats had a femoral sciatic nerve block with 1 mg/kg bupivacaine (Mercury Pharmaceuticals Ltd.) and one cat (cat 3) had a lumbosacral epidural with 0.6 mg/kg bupivacaine and 0.2 mg/kg preservative‐free morphine sulfate (Hameln Pharma Ltd.). In all cats, partial tarsal arthrodesis was performed via open reduction and internal fixation by bone plating. A standard medial or lateral approach was made to the tarsus (Piermattei & Johnson, 2004) based on surgeon preference. After joint exposure, the articular cartilage was debrided from the tarsometatarsal and either calcaneoquartal or proximal and distal intertarsal joints, depending on the approach (lateral and medial, respectively). In one cat (cat 3), it was not noted whether the intertarsal joints were burred. In this study, 0.5 mL of feline demineralised bone matrix (Veterinary Tissue Bank Ltd., Brynkinalt Business Centre/Brynkinallt, Wrexham LL14 5NS) was packed into the debrided articular spaces. In each case, a contoured bone plate was applied with either medial or lateral application. Screws in the proximal metatarsals were angled to engage multiple metatarsal bones. Routine incision closure was achieved in all cats.

### Follow‐up

Clinical and radiographic follow‐up assessment was performed 60 to 87 days post‐operatively. Final recheck examination included visual assessment and physical examination, which included assessment for instability, swelling and pain on palpation of the implants or on manipulation of the arthrodesis site. Lameness was classified using a subjective descriptive assessment (mild, moderate or severe weight‐bearing lameness, or non‐weight‐bearing lameness). Outcomes were classified as full function, acceptable function or unacceptable function according to Cook et al. (2010).

## RESULTS

There were two male and three female cats. Four were domestic shorthair cats and one was a Bengal. Median age was 6.4 years (range 1 year 10 months to 15 years 4 months). Median weight was 4.15 kg (range 4.0 to 5.68 kg). In four cats, the injury was associated with the cat being witnessed to catch the affected hindlimb in a horizontal drawer handle when jumping down from a height, and in one cat, the injury resulted from a dog bite. Three cats were presented with non‐weight‐bearing lameness on the affected limb, while two exhibited marked weight‐bearing lameness. Four cats were referred after a period of conservative management with exercise reduction and analgesia (meloxicam (Loxicom; Norbrook) ± gabapentin (Gabapentin; Noumed Life Sciences Limited)). One cat was referred after treatment with external coaptation in addition to analgesia for 17 days. The mean time from injury to referral was 8.5 days (range 4 to 17 days). Signalment, injuries, treatment, complications and outcome are summarised in Table 1.

**Table 1:** Summary data for five cases of tarsometatarsal instability treated with partial tarsal arthrodesis

Cat	Signalment	Weight	Injury	Treatment	Implants	Complications	Follow‐up (days)	Osseous bridging (%) on radiographs	Outcome
1	15 yr 4 mo MN DSH	4.46 kg	Caught hindlimb in drawer handle, left TMT subluxation, chip fracture to the dorsal aspect of the TMT joint	Laterally plated partial tarsal arthrodesis	7‐hole 1.5/2.0 mm thin locking compression plate (DePuy Synthes). Two 2.0 mm locking screws and one 2.0 mm cortical screw proximal to the TMT joint. One 2.0 mm cortical screw and three 1.5 mm cortical screws distal to the TMT joint	Post‐operative paw swelling (duration not recorded)	65	75	No lameness. Full pre‐operative activities not reached at time of final follow‐up
2	2 yr 4 mo FN DSH	4.12 kg	Caught hindlimb in drawer handle, right TMT subluxation with medial instability, chip fracture of the proximodorsal aspect of a metatarsal bone	Laterally plated partial tarsal arthrodesis	7‐hole 1.5/2.0 mm thin locking compression plate (DePuy Synthes). Two 2.0 mm locking screws and one 2.0 mm cortical screw proximal to the TMT joint. Four 1.5 mm cortical screws distal to the TMT joint	Post‐operative paw swelling (duration not recorded), iatrogenic tarsal valgus associated with imperfect plate contouring	77	50	No lameness. Full return to pre‐operative activities
3	6 yr 5 mo FE DSH	4.0 kg	Attacked by dog, left TMT subluxation with medial instability	Medially plated partial tarsal arthrodesis	9‐hole 1.0/1.5 mm DCP‐style pancarpal arthrodesis plate (Veterinary Instrumentation). Three 1.5 mm cortical screws proximal to the TMT joint. One 1.5 mm cortical screw and four 1.0 mm screws distal to the TMT joint	Post‐operative paw swelling (duration not recorded), loosening and backing out of the most proximal plate screw on radiographs 60 days post‐operatively	60	25	No lameness. Full return to pre‐operative activities
4	1 yr 10 mo FN DSH	4.15 kg	Caught hindlimb in drawer handle, right TMT subluxation with medial instability	Medially plated partial tarsal arthrodesis	6‐hole 1.5/2.0 mm thin locking compression plate (DePuy Synthes). Three 2.0 mm locking screws proximal to the TMT joint. Three 1.5 mm cortical screws distal to the TMT joint	None	87	100	Mild weight bearing lameness. Full return to pre‐operative activities
5	8 yr 1 mo MN Bengal	5.68 kg	Caught hindlimb in drawer handle, right TMT luxation, subjective subluxation of the left intertarsal joint (second to third and first to third), complete fracture of the proximolateral epiphysis of the metacarpal bone V	Laterally plated partial tarsal arthrodesis	9‐hole 1.5/2.0 mm pancarpal locking plate (Veterinary Instrumentation). Four 2.0 mm cortical screws proximal to the TMT joint. Four 1.5 mm cortical screws distal to the TMT joint	Post‐operative paw swelling (duration not recorded)	63	25	No lameness. Full pre‐operative activities not reached at time of final follow‐up

Yr Year, mo Months, MN Male neutered, FN Female neutered, FE Female entire, DSH Domestic shorthair, TMT Tarsometatarsal

All cats were treated with partial tarsal arthrodesis stabilised with a bone plate (Table 1). Medial tarsal instability was identified on pre‐operative assessment in three cats, of which two were plated medially (cats 3 and 4) and one was plated laterally (cat 2). No lateralisation of instability was identified in the remaining two cats (cats 1 and 5), in which the bone plate was applied laterally. Articular cartilage was debrided using a 1.8 mm spinal burr (Veterinary Instrumentation, Distington House, Sheffield, UK) in three cats (cats 1, 2 and 5). Spinal burr size was not recorded in two cats. Demineralised bone matrix was packed into the debrided articular spaces in all cats. No intra‐operative complications were reported.

All cats were weight bearing on the operated limb the day after surgery and were at this stage receiving 0.05 mg/kg oral meloxicam once daily and 0.2 mg/kg intravenous methadone (Comfortan; Dechra Veterinary Products) every 4 hours, which was stopped and replaced with 0.02 mg/kg intravenous buprenorphine (Buprevet; Virbac Ltd.) every 6 hours within 24 hours of surgery in all cats. Four out of five cats (cases 1, 2, 4 and 5) were also receiving 12 to 22 mg/kg of oral gabapentin every 8 to 12 hours immediately post‐operatively. Four of the five cats exhibited post‐operative paw swelling on the operated limb, which remained present at the time of discharge. The duration of paw swelling was not recorded in any cat, but had fully resolved in all cats without treatment by the time of the post‐operative assessment at 60 to 87 days post‐surgery. No other immediate post‐operative complications were noted in any cat.

All cats were discharged mean of 1.6 days post‐operatively (range 1 to 2 days). All cats were discharged with oral gabapentin at a dose of 12 to 22 mg/kg every 8 to 12 hours for 7 days and oral meloxicam at a dose of 0.05 mg/kg once daily for 5 to 32 days. Post‐operative exercise management included crate restriction until the post‐operative recheck appointment. Three out of five cats were permitted to have short periods outside of the crate provided they were under close supervision and not able to run or jump on and off surfaces.

Post‐operative assessments were performed in all cats, a median of 65 days after discharge (range 60 to 87 days). There had been no reported complications between discharge and the post‐operative assessment in any cat. Four of the five cats exhibited no lameness. One cat exhibited a mild weight‐bearing lameness on the operated limb but with no associated pain or instability. No cause of lameness was identified on examination or radiographs, and no further treatment was pursued. One cat had mild iatrogenic tarsal valgus associated with incorrect contouring of the laterally applied plate. Follow‐up radiographs in one cat demonstrated medial displacement of the proximal screw but no other evidence of implant loosening, for which no intervention was pursued. Radiographs in all cats demonstrated progressive fusion of the arthrodesis site, scored as 25% to 100% based on the scoring system of Longo et al. (2025).

## DISCUSSION

Tarsal instability in cats is typically associated with trauma, with previously reported causes including motor vehicle accidents, high‐rise trauma and dog bites (Alza Salvatierra et al., 2018; Schmökel et al., 1994; Yardımcı et al., 2016). Interestingly, in four of the five cats in this study, tarsometatarsal subluxation was associated with the cat catching its paw in a horizontal (kitchen) drawer handle, highlighting a risk for cats descending from raised surfaces where such handles are positioned on their vertical descent path. To the authors’ knowledge, this cause of injury has not previously been described in cats. A similar tarsometatarsal injury has previously been reported in dogs, whereby they catch their hindlimb in a gate or fence while jumping over it (Dyce et al., 1998).

All cats underwent partial tarsal arthrodesis stabilised with a bone plate; three were plated laterally and two medially. Medial tarsal instability was identified in three cats; two of these were plated medially (cats 3 and 4) and one (cat 2) was plated laterally. The remaining two cats (cats 1 and 5) showed no lateralisation of instability and were plated laterally. It has previously been proposed that placement of the plate on the most unstable side of the tarsus may facilitate anatomic reduction and rigid fixation of the injury in patients with tarsometatarsal subluxation (Muir & Norris, 1999). However, lateral application of the bone plate is thought to be technically easier in the authors’ opinion due to increased bone length for plate application (for which the calcaneus can be incorporated).

Successful partial tarsal arthrodesis is reported to give good to excellent outcomes for alleviation of discomfort and return to athletic ability in dogs following tarsometatarsal injuries in the majority of dogs (Dyce et al., 1998; Fettig et al., 2002; Longo et al., 2025; Scrimgeour et al., 2012). A previous study of partial tarsal arthrodesis in working farm dogs reported that 83% of dogs treated for tarsometatarsal injuries were able to return to all or most working duties (Scrimgeour et al., 2012), while two other studies reported 100% of dogs returned to full soundness (Dyce et al., 1998; Fettig et al., 2002). Longo et al. (2025) reported unacceptable function in 20% of dogs with tarsometatarsal injuries treated with partial tarsal arthrodesis. Implant removal was required in 0% to 30% of dogs to achieve soundness (Dyce et al., 1998; Fettig et al., 2002; Longo et al., 2025; Scrimgeour et al., 2012). However, there is limited feline literature regarding outcomes following partial tarsal arthrodesis for tarsometatarsal injuries. No lameness was reported at final follow‐up in four cats in the literature treated with plated partial tarsal arthrodesis (Chow & Balfour, 2012; Inauen et al., 2009; Philips & Jerram, 2025).

The outcomes in this study are comparable to previous reports, with three of the five cats returning to full function based on the criteria described by Cook et al. (2010). Two cats were still having crate restriction when unsupervised, with a plan to return to normal activities after follow‐up assessment. One cat demonstrated mild ongoing lameness but had returned to full pre‐operative activities. No cause for this was identified and no further treatment was pursued. Dyce et al. (1998) reported that 30% of dogs following partial tarsal arthrodesis for tarsometatarsal instability had continued lameness or poor exercise tolerance 3 to 5 months post‐operatively that resolved following implant removal, with no reported evidence of associated infection. Implant removal was not pursued in this case, but it is possible that implant‐related discomfort may have been responsible for the presenting lameness.

Major and minor complication rates of 13% to 22% and 50% to 51%, respectively, are reported for partial tarsal arthrodesis with bone plate fixation in dogs (Muir & Norris, 1999; Roch et al., 2008), with the majority of minor complications being related to external coaptation (Muir & Norris, 1999; Roch et al., 2008). Previous case reports of individual cats (Bright et al., 2007; Inauen et al., 2009), two cats in a larger case series of tarsal injuries (Schmökel et al., 1994) or two cats in a larger study on carpal and tarsal arthrodesis in dogs and cats (Théoret & Moens, 2007) reported no complications in any cats following feline partial tarsal arthrodesis with plate stabilisation. Requirement for explantation of the lateral plate following a biaxially plated partial tarsal arthrodesis due to surgical site infection is reported in one cat (Philips & Jerram, 2025). No major or catastrophic complications were reported for any cat in this series within the follow‐up period undertaken. Minor complications were post‐operative paw swelling in three cats and mild tarsal valgus in one cat. Neither complication was considered to be clinically significant.

Post‐operative paw swelling occurred in three cats where post‐operative external coaptation was not used, but did not occur in one cat where external coaptation with a soft padded bandage was used for 1 day post‐operatively. The time to resolution of swelling was not noted for any of these cats. Distal limb swelling is reported in 9.6% of dogs following partial tarsal arthrodesis with bone plate fixation (Roch et al., 2008), and in the author’s experience, it is not uncommon. Mild tarsal valgus occurred in one cat due to imperfect contouring of the bone plate with no associated lameness. Iatrogenic tarsal valgus or varus deformity has previously been reported in seven dogs following partial tarsal arthrodesis with plate stabilisation, representing 20% to 30% of cases (Dyce et al., 1998; Longo et al., 2025; Scrimgeour et al., 2012). In working farm dogs, two out of four with iatrogenic valgus continued to work, while two were unable to return to their previous activities or perform most duties, even if allowance was made for reduced performance (Dyce et al., 1998; Scrimgeour et al., 2012). Full function was reported in two of three dogs with varus or valgus deformity following plated partial tarsal arthrodesis, with the remaining dog having acceptable function for which the working status was not reported (Longo et al., 2025).

This study has several limitations. The number of cases was small despite the long study period, and the retrospective design necessitated reliance on the accuracy and completeness of the clinical records, which may have led to under‐reporting of complications. Multiple surgeons were involved over the 13‐year period, introducing variability in surgical technique, perioperative management and record keeping. Owing to the terms of ethical approval, only retrospective review of hospital records was permitted, and follow‐up by direct contact with owners or referring veterinarians was not allowed. Therefore, complications developing after the final recorded hospital visit may not have been captured. Assessment of post‐operative outcome was based on subjective owner‐reported return to function and analgesia requirements, and lameness was graded descriptively in the clinical notes rather than using a standardised scoring system or objective gait analysis (e.g. force‐plate or walkway data). The previously described method for assessing tibio‐tarso‐metatarsal alignment (Longo et al., 2025) could not be applied because the available post‐operative radiographs were not collimated to include the anatomical landmarks required for this measurement. Consequently, radiographic alignment was assessed subjectively.

The results of this study suggest that partial tarsal arthrodesis with bone plate fixation is a successful method for the management of traumatic subluxation of the tarsometatarsal joint in cats. It is commonly associated with minor paw swelling in the immediate post‐operative period. It is associated with excellent outcomes for return to pre‐injury activities.

## Conflict of interest

The authors have no conflict of interest to declare.

## Data Availability

The data that support the findings of this study are available from the corresponding author upon reasonable request.
